# Immunization induces inflammation in the mouse heart during spaceflight

**DOI:** 10.1186/s12864-025-11426-y

**Published:** 2025-03-10

**Authors:** Alicia L. Veliz, Lorelei Hughes, Delia Carrillo, Michael J. Pecaut, Mary Kearns-Jonker

**Affiliations:** 1 Department of Pathology and Human Anatomy, Loma Linda University School of Medicine, Loma Linda, CA 92350 USA; 2 Department of Basic Sciences, Loma Linda University School of Medicine, Loma Linda, CA 92350 USA

**Keywords:** Spaceflight, Microgravity, International space station, Transcriptomics, NF-κB, Immunization, Myocarditis, Inflammation, Heart, Cardiovascular system

## Abstract

Space travel is a growing area of interest and includes initiatives such as NASA’s Moon-to-Mars Mission. Reports on the cardiovascular effects of space travel reveal changes in morphology, metabolism, and function of the cardiovascular system. In this study, the cardiovascular response to immunization in space was studied in mice which were housed and immunized while on the International Space Station (ISS). Mice were immunized with tetanus toxoid combined with the adjuvant CpG (TT + CpG) and the effects of vaccination in space were studied using transcriptomics. Analysis of the mouse heart transcriptome was performed on flight control and flight-immunized mice. The results show that immunization aboard the ISS stimulates heightened inflammation in the heart via induction of the nuclear factor kappa B (NF-κB) signaling pathway to promote the release of the pro-inflammatory cytokines IFNγ, IL-17 and IL-6. Additional transcriptomic changes included alterations in the cytoskeleton and in the expression of transcripts associated with protection from oxidative stress. In summary, inflammation in the heart can occur following immunization in space. This investigation explores the impact of immune challenges on the heart and lays the groundwork for future research into additional cardiac alterations which can occur during spaceflight.

**Supplementary Information:**

The online version contains supplementary material available at 10.1186/s12864-025-11426-y.

## Background

The effects of microgravity and space-related radiation have the potential to impact astronaut health on long-duration missions. Indeed, human and animal model studies have already shown structural and/or functional deficits in the musculoskeletal system, the lymphatic and blood circulatory systems, and immune system after spaceflight [1–3]. Of particular concern is the impact of spaceflight on the cardiovascular system. It is already well known that microgravity leads to significant fluid shifts and subsequent decreases in total blood volume, potentially changing physiological stressors inherent to normal heart function [4, 5]. Astronauts experience dysregulation of their lymphatic and blood circulatory systems while in space, resulting in a shift of fluids towards the head [5, 6]. Some investigators have also linked both microgravity and the space radiation environment to increased risk for cardiovascular disease [7, 8].

Inflammation is a well-known component of cardiovascular disease in ground-based studies [9, 10]. However, the link between inflammation and spaceflight-induced changes in cardiac function has not been extensively studied. Our previous work has demonstrated that the spaceflight environment does not induce cardiac inflammation in mice which were housed on the ISS for 30 days [11]. This is surprising as it has long been known that spaceflight can increase inflammatory markers in many tissues, including the musculoskeletal system, liver and kidneys [12–14]. In rare cases, immunization can induce myocarditis associated with cardiac inflammation [15–18]. We therefore sought to determine whether or not the stress of the spaceflight environment exacerbates the risk for vaccine-induced cardiac inflammation, increasing the risk of cardiovascular disease in astronauts.

To explore the impact of combined physiological and immunological stressors on cardiovascular risk, RNAseq analysis was used to identify transcriptomic changes in the hearts of mice after immunization in space. Our results showed that immunization during spaceflight activated an inflammation-related gene expression response in the heart similar to myocarditis on Earth.

## Materials and methods

### Experimental animal model

Animal experiments were reviewed and approved by the NASA Flight Institutional Animal Care and Use Committee (FIACUC) (protocol #RR-12). The experiments described here were a component of a larger investigation which was focused on the impact of immunization in the context of the spaceflight environment. The current study was designed to investigate the effects of immunization on the heart in space. Heart tissue was provided to our laboratory by the NASA Biospecimen Sharing Program. Eight- to nine-week-old female C57BL/6J mice (Jackson Laboratory, Bar Harbor, ME) were delivered to the Kennedy Space Center (KSC) Animal Care Facility. Due to the limited number of mice that could be approved for housing on the ISS and to facilitate the interpretation of the data, the inbred mice in all of the experimental and control groups were selected to be of the same age and sex. This allowed us to distribute a uniform set of mice of the same strain and sex into the various groups. Mice were acclimatized for 1 to 2 weeks before study initiation. During this period, their living conditions were controlled for temperature and humidity, and a 12:12 h light: dark cycle was established.

Treatments were assigned randomly for each cage. The body mass of the mice within each of the cages and across groups was measured to ensure that there were no statistically significant differences in body mass within and between the groups of flight and ground control mice. The entire cage of mice was not used for the project if any mice in the cage showed signs of stress, such as over-grooming or fighting during the adaptation period, in order to maintain consistency across all treatment and control groups. Complete cages were then divided into one of three treatment groups (Saline, TT + CpG, CpG only) for ground controls and flight mice. All five mice within one cage received the same treatment. For the purpose of this study, we focused on thirty mice, as indicated in Table 1, in order to identify the effects of immunization with tetanus toxoid plus adjuvant in the context of spaceflight.

**Table 1:** Animal numbers in flight and control groups

Group	Treatment	Total *N* number per group	Samples used for RT-qPCR	Samples used for Sequencing
Flight	TT + CpG	5	5	3
	Saline	5	5	3
	CpG only	5	3	−
Ground	TT + CpG	5	5	−
	Saline	5	5	−
	CpG only	5	3	−
Total: *n* = 30				

Ground controls remained at the Kennedy Space Center (KSC) in the ISS Environmental Simulator (ISSES), a controlled environment chamber that matched temperature, humidity and CO_2_ levels from the ISS on a 48-hour delay via telemetry. Flight groups were flown to Wallops Island (VA), placed on board the Northrop Grumman robotic resupply spacecraft Cygnus mission 11 (NG-11), and sent to the International Space Station (ISS).

To ensure a robust immune response while in space, mice were first vaccinated on the ground 13 days prior to launch with an intraperitoneal (IP) injection of either 0.1 ml saline vehicle (control), 0.1 ml of 10Lf/ml tetanus toxoid (AJ Vaccines, Denmark) + 0.1 ml of 0.8 mg/ml CpG ODN 1826 adjuvant (InvivoGen), or + 0.1 ml of 0.8 mg/ml CpG ODN 1826 adjuvant only, allowing time for the development of immunological memory. A second, identical vaccination was given to the flight mice 21 days after launch. Saline was administered to flight control animals. Finally, the flight mice were euthanized via IP injection of ketamine/xylazine (3 mg/0.9 mg ketamine/xylazine in a volume of 300 μl PBS) 13–14 days after the second immunization. The euthanized mice were wrapped in foil and kept frozen at -76 °C until they were returned to Earth. Hearts were later extracted from semi-thawed animals, flash-frozen and stored at -80 °C until RNA was prepared for transcriptomics. Comparable ground treatment (TT+CpG, CpG only) and control (saline) mice were maintained at the Kennedy Space Center.

### RNA and cDNA synthesis

To extract RNA, < 25 mg of tissue was cut on dry ice and the tissue was collected and suspended in QIAzol® Lysis Reagent (QIAGEN, Germantown, MD). Samples were then homogenized, and RNA was extracted using a miRNeasy Mini Kit (QIAGEN, Germantown, MD). Sample quality was assessed with gel electrophoresis and Nanodrop. cDNA was synthesized using Superscript III and the manufacturer’s recommended protocol (Invitrogen, Grand Island, NY).

### RNA sequencing and transcriptomic analysis

The RNA samples for three spaceflight saline-injected control mice and three spaceflight mice which were immunized with TT + CpG, were shipped to LC Sciences (Houston, TX, USA) on dry ice for RNA sequencing followed by bioinformatic analysis. RNA with a RIN above 8 (Agilent Technologies 2100 Bioanalyzer) was used to generate a sequencing library. The poly(A) RNA sequencing library was made following Illumina’s TruSeq-stranded-mRNA sample preparation protocol. Quality control was done using Agilent Technologies 2100 Bioanalyzer High Sensitivity DNA Chip. Sequencing was performed on Illumina NovaseqTM 6000 following the vendor’s protocol.

To analyze the RNA-seq data, the workflow involved using in-house Perl scripts in addition to Cutadapt (version 1.10) for trimming any adapter sequences and processing the raw sequence data [19]. A thorough assessment using FASTQC (version 0.10.1) was performed and the trimmed reads were aligned to the reference mouse genome (Ensembl release-101 mus_musculus; v101) using HISAT (version 2.0) [20]. Gene assembly was conducted by calculating the expression of genes based on the total number or coverage of reads aligning to each gene locus. Full-length transcripts and splice variants were assembled and quantitated using StringTie [21]. Individual transcriptomes were combined to create a complete transcriptome. This was done by merging transcripts using Perl scripts and GffCompare. Stringtie (version 1.3.4) and ballgown (http://www.bioconductor.org/packages/release/bioc/html/ballgown.html; version 3.20) were then used to approximate the expression levels of each completed transcript. StringTie was utilized to quantify mRNA expression levels across all six mice, as represented by the Fragments Per Kilobase of transcript per Million mapped reads (FPKM) metric. Transcripts with a false discovery rate (FDR) below 0.05 and an absolute fold change ≥ 2 were considered differentially expressed. Differential expression analysis was performed using DESeq2 and edgeR [22, 23]. These tools used a negative binomial distribution within a generalized linear model (GLM) framework for modeling RNA sequencing count data to identify significant changes in gene expression. This was followed by GO (2019.05) and KEGG (2019.05) enrichment analyses [24, 25]. Single Nucleotide Polymorphism (SNP) and insertion-deletion (Indel) analyses were carried out using SAMtools (version 0.1.19), and variant annotation was conducted with ANNOVAR (2017.09). For alternative splicing analysis, rMATS (version 4.1.1) was employed for use of Gene Set Enrichment Analysis.

Differentially expressed genes and transcripts were uploaded for analysis using Ingenuity Pathway Analysis (IPA, QIAGEN), Gene Set Enrichment Analysis (GSEA), and DAVID Bioinformatics [26–29]. These programs analyze large-scale omics data to determine changes in pathway activation and biological function. Three flight immunized and three flight saline hearts were included in the transcriptomic analyses. All software used for RNA sequencing and downstream analysis is listed in Supplementary Tables S1 and S2.

### RT-qPCR

RT-qPCR was used to validate transcriptomic findings comparing the flight control and the flight TT + CpG, as well as comparable ground controls. In addition, administration of CpG only versus immunization with TT + CpG was assessed using quantitative PCR. Primer pairs were designed using NCBI PrimerBlast (Table S3). GAPDH was used as a housekeeping gene. RT-qPCRs were run at 94^o^C for 10 min, 94^o^C for 15 s, 56-58^o^C (depending on the primer) for 60 s and 72^o^C for 30 s for 45 cycles. Results were analyzed using the delta delta cycle threshold (ΔΔCT) method for relative gene expression [30]. Five mouse hearts were analyzed in each ground control group as well as in each of the flight immunization and control mouse groups. Three to five technical replicates were used for each RT-qPCR analysis. Values for the flight-immunized group were normalized to their respective flight saline control group and ground-immunized group. To assess the normality of values, we conducted a Shapiro-Wilk test. If the values successfully passed this test, we proceeded with an unpaired t-test to determine their statistical significance. In the event that non-normality was detected in any dataset, we utilized Wilcoxon’s t-test to analyze their statistical significance. T-tests were performed on Graphpad’s Prism at a significant *p-value* less than 0.05 (*=*p* < 0.05, **=*p* < 0.01, ***=*p* < 0.001, ****=*p* < 0.0001).

## Results

### The mouse cardiac transcriptome after immunization on the ISS

**Fig. 1:**
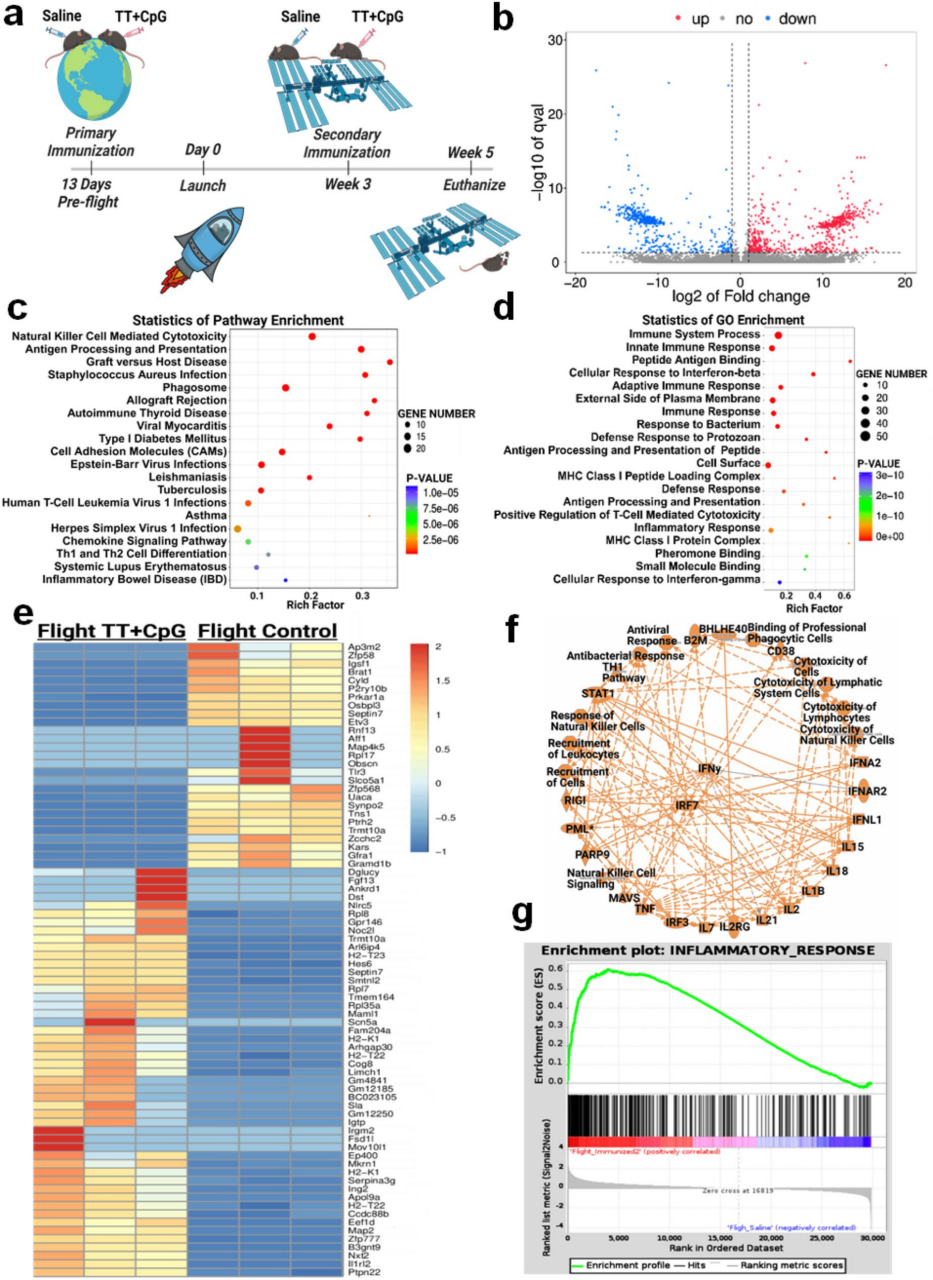
The Impact of Tetanus Toxoid + CpG Immunization on the Heart in Space. (**a**) Two groups of mice were sent to the ISS for 34–35 days. One group was vaccinated with tetanus toxoid plus CpG 13 days before launch, and again after 21 days in space. A second group, vaccinated with saline only, served as a control (created with BioRender.com). Transcriptomic analysis was conducted with *n* = 3 biological replicates per group. (**b**) The volcano plot identified significant differences in the flight-immunized mice in reference to the flight control. The red clusters show a positive change, and the blue clusters show a negative change in transcript expression compared to spaceflight controls. (**c**) GO Enrichment analysis of statistically significant transcripts (*p* < 0.05) shows the enriched processes in flight-immunized mice versus flight saline mice. (**d**) KEGG analysis revealed that the statistically significant transcripts promoted an immune response and inflammation in the heart. (**e**) The heatmap lists the top 80 up- and down-regulated genes in the flight-immunized mice when compared to the flight-saline mice. Many highly regulated genes in flight-immunized mice (left three panels) were focused on immune-associated markers when compared to flight control mice (right three panels), where, in the heat map, red shows upregulation and blue downregulation in Fragments Per Kilobase Million. (**f**) Ingenuity Pathway Analysis of all statistically significant, differentially expressed transcripts predicted increased cytokine signaling as well as recruitment and response of immune cells (orange = predicted activation, blue = predicted downregulation). (**g**) Gene Set Enrichment Analysis (GSEA) shows that the inflammatory response was upregulated in the flight-immunized mice when compared to flight saline (*FDR q-value = 0.0*)

The transcriptomic changes induced in the heart following vaccination with tetanus toxoid plus the adjuvant CpG versus flight saline were identified using RNAseq (*n* = 3 per group) (Fig. 1a). The hearts of mice vaccinated in flight showed 658 upregulated and 472 downregulated transcripts (*p* < 0.01) when compared to flight saline mice (Fig. 1b). Gene Ontology (GO) Enrichment and Kyoto Encyclopedia of Genes and Genomes (KEGG) analysis on all significantly expressed transcripts indicated that inflammatory and immune responses were most highly activated (Fig. 1c, d). A comparative overview of differential transcript expression in flight-immunized mice and flight saline mice is shown in the heat map (Fig. 1e). The data collected from RNA sequencing was uploaded to Ingenuity Pathway Analysis (IPA) to assess the impact of immunization in space on major biological processes in the heart. A graphical summary of the regulation of these processes is shown in Fig. 1f. Interferon regulatory factor 7 (IRF7), interferon-gamma (IFNγ), interferon alpha 2 (IFNα2), signal transducer and activator of transcription 1 (STAT1), and interleukin 2 (IL-2) were predicted to be activated (orange). These signaling transcripts promote immune responses and enhance pathway activation. Similarly, Gene Set Enrichment Analysis (GSEA) identified an upregulation of the overall inflammatory response (Fig. 1g).

### Toll-like receptor-mediated activation of cardiomyocytes

**Fig. 2:**
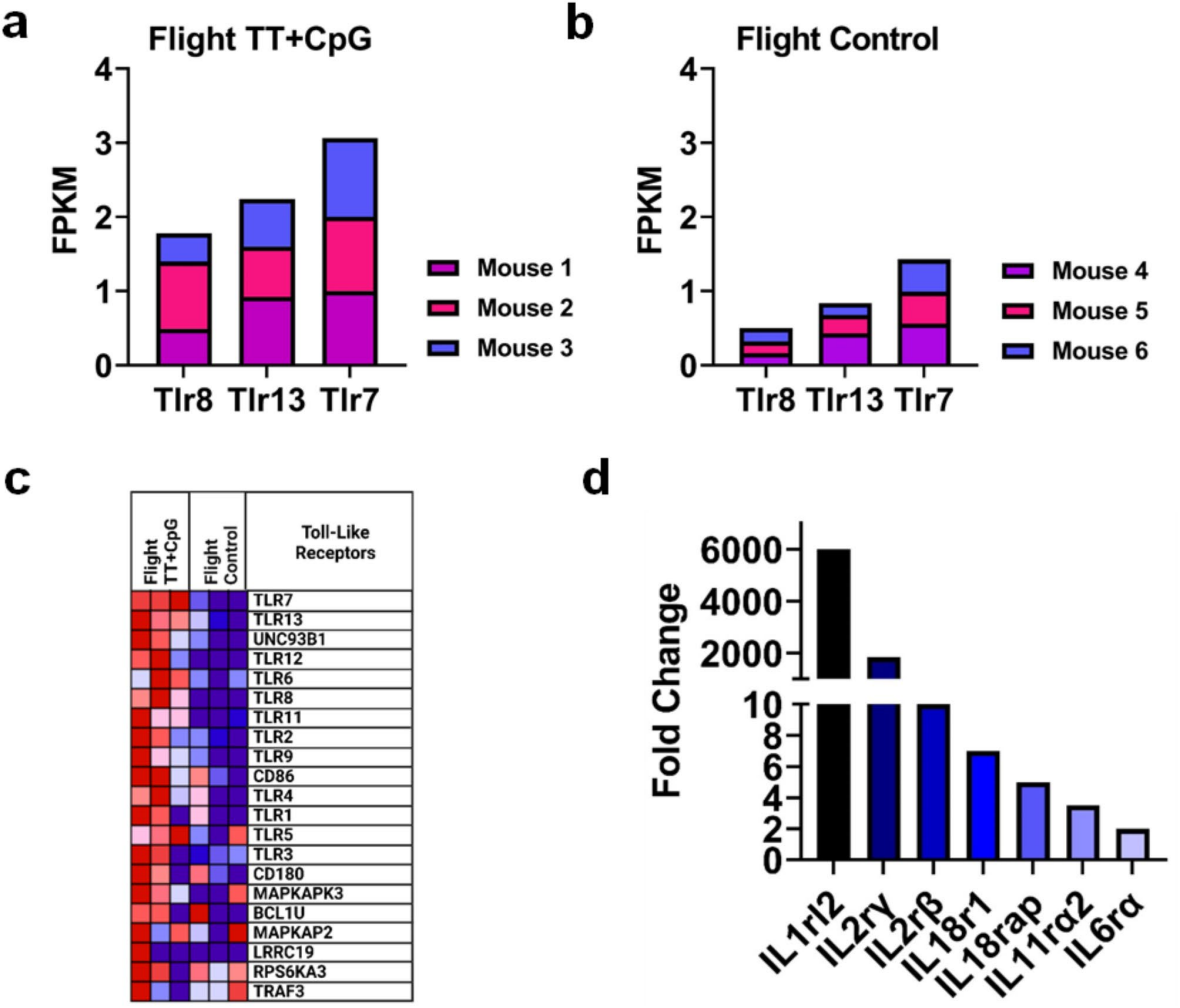
Genes Encoding Toll-Like Receptors are Induced in the Heart after Immunization in Space. **a**, **b**) Transcriptomic analysis showed induction of genes for Toll-like receptors 7, 8, and 13 following immunization in spaceflight when compared to flight control. **c**) Gene Set Enrichment Analysis (GSEA) showed enrichment of genes associated with Toll-like receptor pathways. The enriched genes were depicted in red, while the genes that were not enriched were shown in blue. In this representation, the relative intensity of the color indicates the strength of gene enrichment (e.g., saturated red versus light red) or decreased gene enrichment (e.g., saturated blue versus light blue) **d**) Receptors for interleukins 1, 2, 6, 11 and 18 were upregulated in flight-immunized mice when compared against flight control mice. Transcriptomic analysis was conducted with *n* = 3 biological replicates per group

Toll-like receptors (TLRs), or pattern recognition receptors, were analyzed for increased expression following vaccination. The adjuvant used in this study, CpG ODN 1826, is recognized by Toll-like receptor 9 (TLR9) [31]. However, vaccination with TT + CpG in space showed a trend toward elevated expression of several other TLRs in cardiovascular tissue. In flight-immunized mice, there was a significant increase in expression of Toll-like receptors 7, 8, and 13 when compared to the flight control group. (Fig. 2a, b). Gene Set Enrichment Analysis (GSEA) identified enrichment of Toll-like receptors 1–9 and 11–13 (Fig. 2c). Furthermore, there was a significant upregulation of receptors for interleukins 1, 2, 6, 11, and 18 (2 < fold change (fc) < 6,000, *p-value < 0.05*, Fig. 2d).

**Fig. 3:**
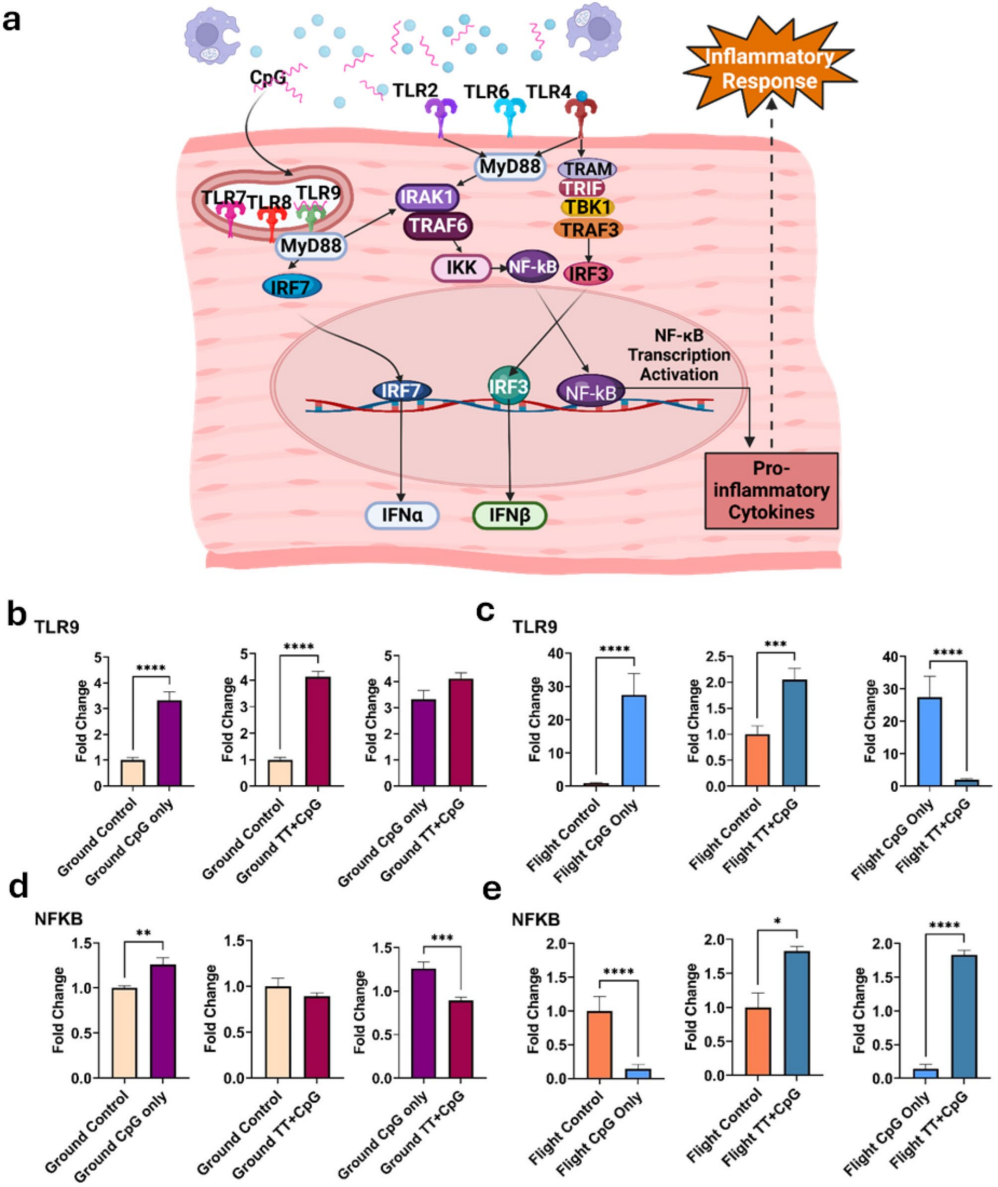
Toll-Like Receptor-Mediated Inflammatory Response in Cardiomyocytes. **a**) NF-κB-dependent transcription of pro-inflammatory cytokines is induced in cardiomyocytes following CpG activation of endogenous Toll-like receptor 9. Downstream effects promote an inflammatory response in cardiac tissue (created with BioRender.com). **b**, **c**) TLR9 expression was significantly upregulated following vaccination of CpG alone in flight, on the ground, and post-TT + CpG administration in flight mice only (*n* = 3–5, 2 < fc < 22, t-test, *p-value < 0.05*). **d**, **e**) NF-κB induction occurred via CpG-only administration on the ground but required combined administration of TT plus CpG in space (*n* = 3–5, fc > 1.5, t-test, *p-value < 0.05*). The flight and ground TT + CpG groups and the flight and ground CpG-only group were each normalized to their appropriate control groups

When CpG activates TLR9, it promotes a cascade of signaling molecules within cardiomyocytes that leads to the translocation of transcription factors IRF7 and NF-κB to the nucleus. Once they enter the cells, they will begin transcription of IFNα and pro-inflammatory cytokines, respectively. This proposed mechanism of inflammation in cardiomyocytes is outlined in Fig. 3a.

On Earth, CpG alone can evoke an immune response through activation of TLR9 and, subsequently, NF-κB (Fig. 3b, d). In spaceflight mice, RT-qPCR was used to show that there was a significant elevation in TLR9 expression following vaccination with CpG alone. However, administration of CpG alone in space was not sufficient to lead to the activation of NF-κB (Fig. 3c, e). In flight, activation of NF-κB only occurred when administration of the adjuvant CpG was combined with tetanus toxoid.

### Inflammatory response induced in the heart after immunization in spaceflight

**Fig. 4:**
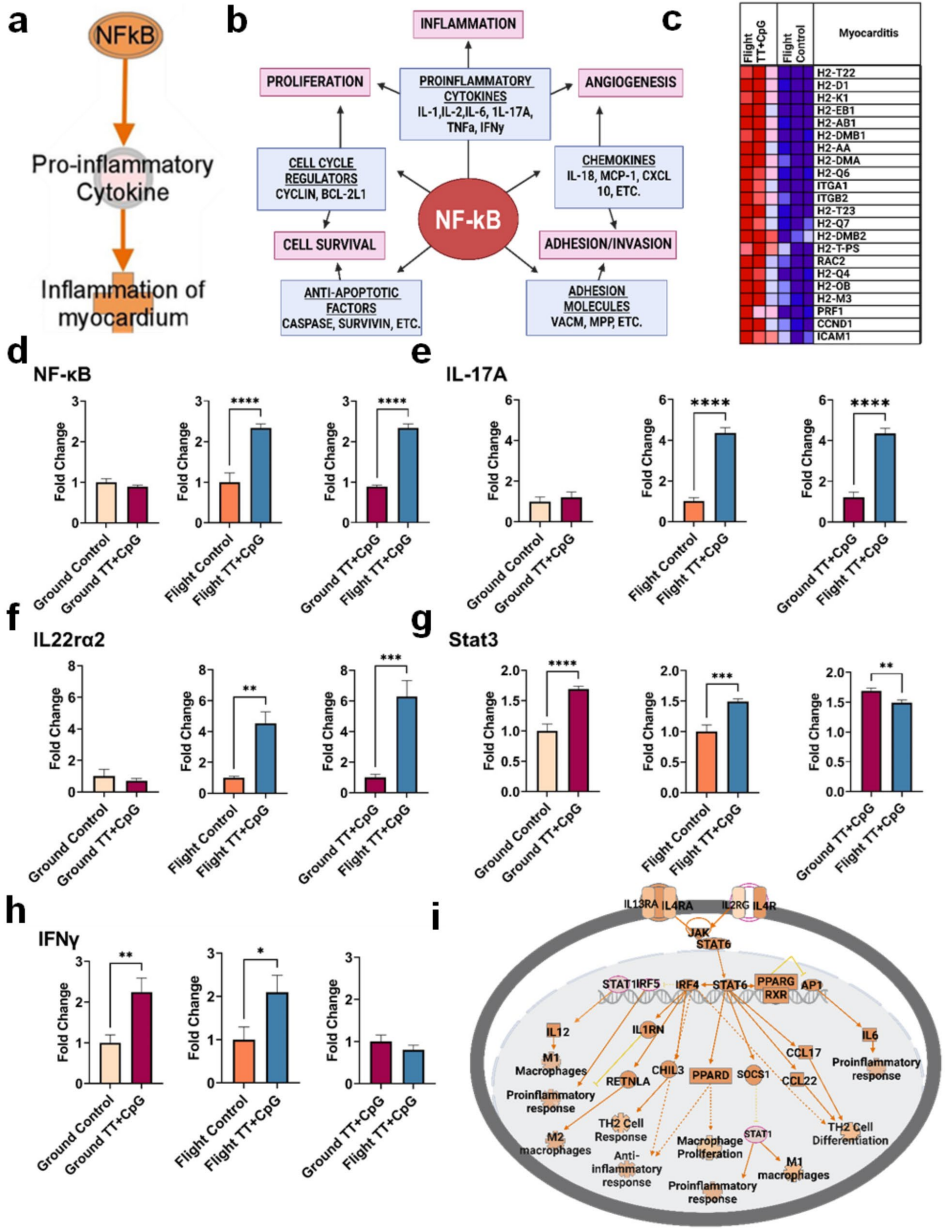
Inflammation Occurs in Mouse Hearts Immunized in Space. (**a**) According to IPA, transcripts that lead to inflammation of the myocardium were predicted to be upregulated in immunized flight mice when compared to saline controls (orange = predicted activation). (**b**) NF-κB is a known regulator of signaling molecules which activate processes such as inflammation (created with BioRender.com). (**c**) Gene Set Enrichment Analysis identified positive regulation of Interleukin 6 production following immunization with TT + CpG in space. **d**-**f**) Markers of inflammation, including NF-κB, IL-17A, and IL22ra2, were evaluated via RT-qPCR and were shown to be activated in the mouse heart post-vaccination (1.5 ≤ fc ≤ 4 in flight-immunized mice, *p-value < 0.05*). **g**) STAT3 was significantly upregulated in all immunized mice when compared to controls (fc > 1, *p-value < 0.05*). **h**) IFNγ, a pro-inflammatory cytokine, was elevated in flight and ground-immunized mice (fc ≥ 2, *p-value < 0.05*). Flight-immunized and ground-immunized groups were each normalized against their own flight or ground control. **i**) Macrophages in the heart of flight-immunized mice were predicted to be activated according to IPA, where upregulation is identified by an orange color and downregulated transcripts are identified in blue (created with BioRender.com). Flight-immunized versus flight-control mice were compared by uploading transcripts which were altered in expression at *p-value < 0.05*

To further our understanding of the effects of immunization in space, we used transcriptomic analysis in combination with RT-qPCR to conduct a targeted analysis. IPA predicted that immunization in flight promoted activation of NF-κB in cardiomyocytes, leading to the production of pro-inflammatory cytokines and, eventually, inflammation of the myocardium (Fig. 4a, Table S4-S5). Gene Set Enrichment Analysis also identified upregulated transcripts that activate the NF-κB signaling pathway (Table S6). Transcription factor NF-κB is involved in inflammatory pathways where increased expression activates and maintains the production of pro-inflammatory cytokines and signaling molecules, such as interleukin 6 (IL-6), a key modulator of chronic inflammation (Fig. 4b). Among the immune-related pathways most activated by vaccination in flight mice, interleukin 6 was one of the most significantly changed. Given the changes in gene expression related to the regulation of IL-6 (Fig. 4c), we focused on transcripts identified by GO enrichment analysis as related to the regulation of inflammatory pathways. Signaling from its receptor, IL6rα (Fig. 2d), promotes the transition of infiltration from neutrophilic to mononuclear, furthering inflammation from acute to chronic [32, 33].

In order to verify that inflammation was upregulated in spaceflight after immunization, likely through an NF-κB-dependent pathway, RT-qPCR analysis was conducted on both flight groups of mice, control and immunized, as well as comparable ground controls. Vaccination led to a significant increase in NF-κB expression in flight-immunized mice, which was not observed in ground-immunized mice (Fig. 4d). Interleukin 17A (IL-17A) and interleukin 22 receptor subunit alpha 2 (IL-22rα2) were similarly induced in the flight-immunized mice, but not in ground-immunized mice (Figure e, f). Signal transducer and activator of transcription 3 (STAT3) and interferon gamma (IFNγ) were elevated in flight and ground-immunized mice (Fig. 4g, h). Cytokines interleukin 1 beta (IL-1β) and tumor necrosis factor alpha (TNF-α) were analyzed by RT-qPCR but were not induced in mice which were immunized in space (Figure S1). Transcripts encoding a subset of cytokines specific to the NF-κB inflammatory pathway were elevated in mice following immunization while housed on the ISS.

Although we did not identify specific cell populations in our bulk transcriptomic analysis, macrophage activation was predicted in the immunized flight mice when compared to their saline-treated counterparts (Fig. 4i). IPA predicted that a pro-inflammatory response of macrophages occurs via IL-6 activation, and polarization of M1 macrophages occurs through interleukin 12 (IL-12) activation. Gene Set Enrichment Analysis identified positive regulation of IL-12 production after TT + CpG vaccination in flight-immunized mice (Figure S1).

### Immunization in spaceflight amplifies cytoskeletal rearrangements

**Fig. 5:**
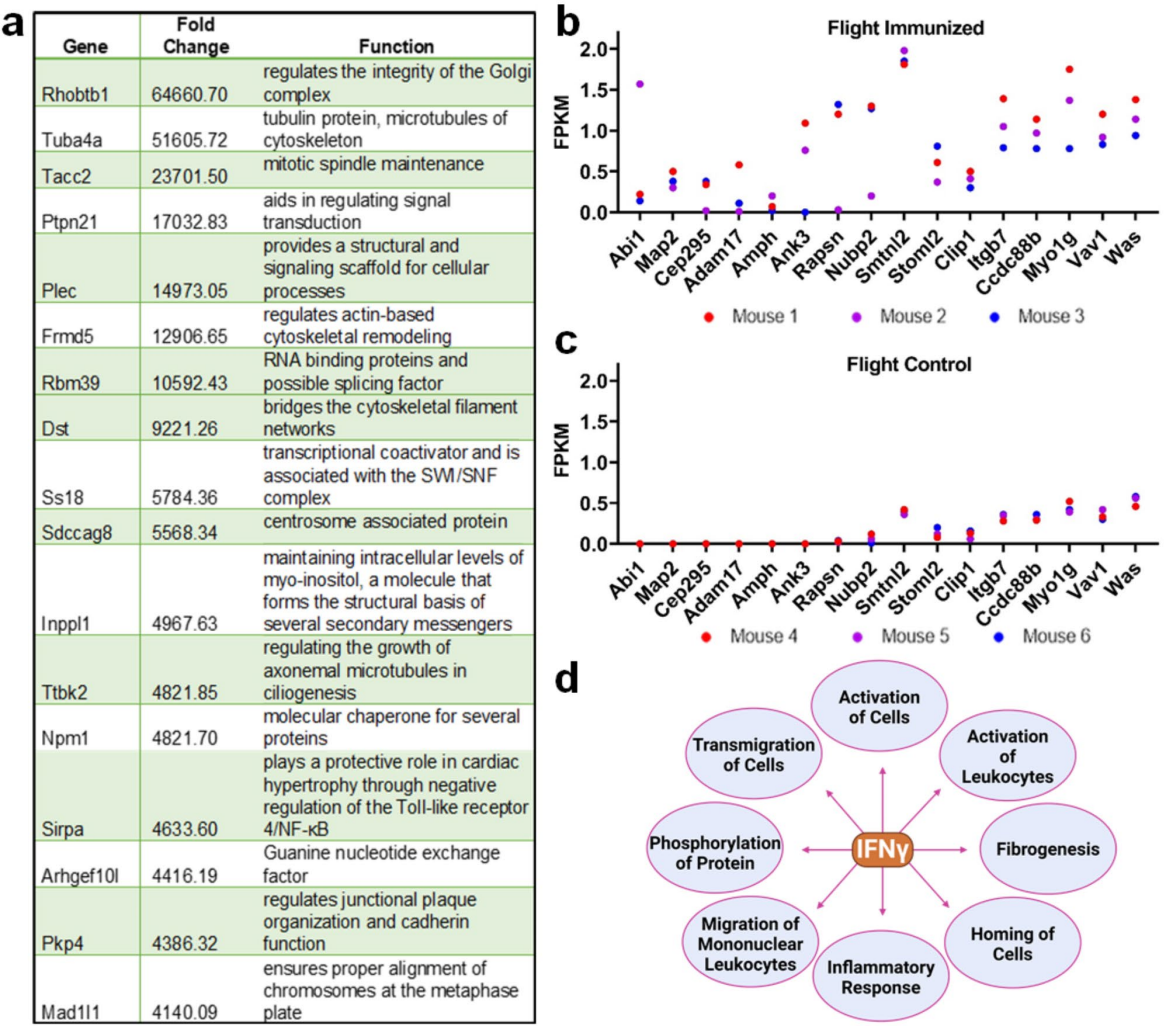
Cytoskeletal Interaction during Immune Response in Space. **a**) Top 18 significantly induced cytoskeletal markers with fold changes above 4,000. **b**, **c**) Analysis of differentially expressed transcripts revealed an upregulation associated with cytoskeletal rearrangement in flight-immunized mice (*p* < 0.05). **d**) Functions of IFNγ following activation in flight-immunized mice (created with BioRender.com). Transcriptomic analysis was conducted with *n* = 3 biological replicates per group

Transcripts related to cytoskeletal rearrangement were significantly induced following immunization in space. Elevated transcripts with fold changes above 4,000 are listed in Fig. 5a, along with their functional role. These transcripts enhance signal transduction and motility between cells and mediate tubulin protein and actin-based cytoskeletal filaments. The cytoskeletal-associated transcription factor signal-regulatory protein alpha (SIRPA) promoted cardiac protection through the regulation of Toll-like receptor 4 and NF-κB. Elevated transcripts in flight-immunized mice as compared to flight-control mice are shown in Fig. 5b and c, in which each distinct color denotes a different mouse in each flight group. The individual data points indicate the differential transcript expression of each of the cytoskeletal-related markers. According to the IPA and GSEA analysis, transcripts related to cytoskeletal rearrangement were elevated following IFNγ activation (Tables S7, S8). This activation promotes leukocyte activation, mononuclear leukocyte migration, cell transmigration, and inflammatory responses (Fig. 5d).

### Transcripts protecting the heart from oxidative stress are induced in spaceflight

**Fig. 6:**
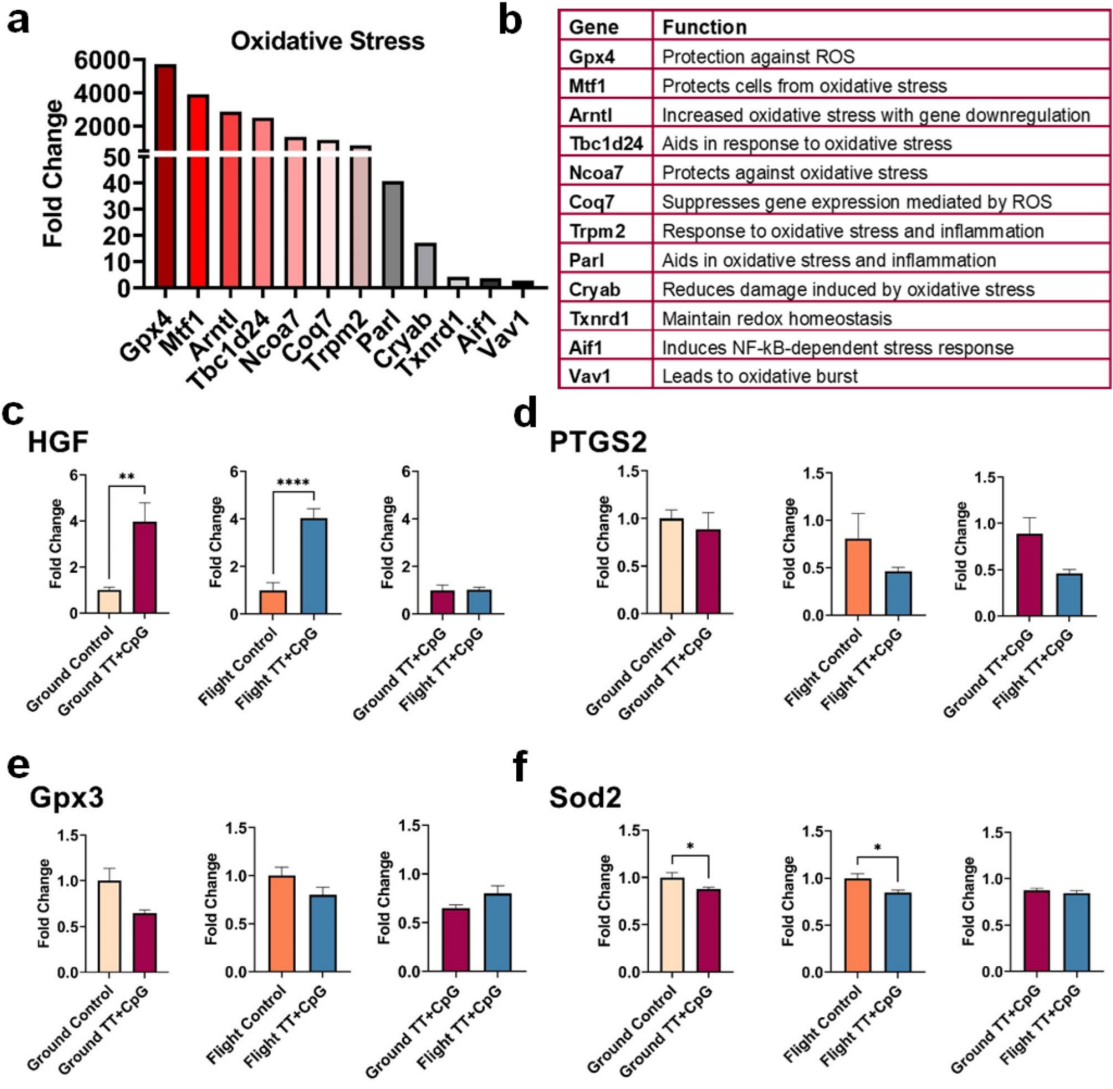
Elevated Expression of Transcripts Protecting the Heart from Oxidative Stress in Flight-immunized Mice. (**a**) Oxidative stress transcripts were significantly elevated in flight mice after immunization when normalized to flight control mice, according to transcriptomic analysis. (**b**) The function of several upregulated transcripts related to oxidative stress included protection from cell damage through increased response to stress and ROS. Many of these markers lead to cell and mitochondrial protection. (**c**) RT-qPCR analysis showed that hepatocyte growth factor (HGF), a cardioprotective gene active during heart damage, was highly elevated in all immunized mice when normalized to their appropriate controls (fc ≥ 4, *p-value < 0.05*). **d**-**f**) RT-qPCR was used to verify that no significant change in common oxidative stress markers was detected in immunized mice when compared to flight control and ground immunized mice (*p-value < 0.05*)

Transcripts that aid in protecting the heart from the effects of reactive oxygen species (ROS) were elevated after immunization in space (Fig. 6a, b). Transient receptor potential melastatin 2 (Trpm2) and thioredoxin reductase 1 (Txnrd1) respond to inflammation by maintaining homeostasis and were upregulated in flight mice after immunization. Many of the protective transcripts induced in this model exhibited a fold change exceeding 1,000 in the flight-immunized mice (*p* < 0.01) (Fig. 6a). RT-qPCR was used to further validate the transcriptomic data. We analyzed hepatocyte growth factor (HGF), which protects various organ systems, including the heart, against the effects of oxidative stress [34–36]. HGF transcript levels were significantly increased in all immunized mice (Fig. 6c). Notably, glutathione peroxidase 3 (Gpx3), superoxide dismutase 2 (Sod2), or prostaglandin-endoperoxide synthase 2 (PTGS2), mediators of oxidative stress through reactive oxygen species stabilization, were not induced in flight-immunized mice (Fig. 6d-f).

## Discussion

In this study, we identified transcriptomic changes that occur in the mouse heart following immunization with TT + CpG in the spaceflight environment. A shift in cytokine expression and activation of the NF-κB pathway, leading to inflammation, occurred following vaccine administration. Although reports of myocarditis associated with vaccination on Earth are rare [37, 38], immunization with an adjuvant under microgravity conditions aboard the ISS can activate transcripts associated with inflammation in the heart.

Tetanus toxoid vaccinations have been implemented as part of health regimes worldwide, commonly as part of the TDAP vaccination series [39]. The tetanus toxoid vaccine contains an inactivated form of the tetanus toxin. This version is recognized by the body, priming immune cells for future encounters. Tetanus is a potentially life-threatening disease commonly found on metal surfaces. Vaccinations have been developed to prevent the bacterial neurotoxin that causes the disease. The tetanus vaccine is first administered at two months of age and is followed by boosters every ten years through adulthood [39]. Our study utilized the tetanus toxin vaccination model since it has the potential to be relevant for astronauts on long-duration space missions. Accordingly, vaccinations and boosters for other diseases should be studied in the future for prolonged missions. It has recently been reported that thirteen strains of *E. bugandensis*, a drug-resistant bacterium, were isolated from the ISS [40]. Under stress, mutations in these strains occurred such that they evolved to become distinct compared to their Earth counterparts [40]. Astronauts may require immunization while in space due to these or other potential bacteria, including *Clostridium tetani*, the bacteria that causes tetanus [41]. Understanding how space changes the response to pathogens is important, especially because vaccination in flight elicits a dampened immune response when compared to vaccination on Earth [42, 43].

Changes in the physical environment can disrupt cell function and cell signaling. Immune cells readily respond to cytokine signaling. Microgravity produces a shift in the expression of cytokines and their receptors [44–46]. In space, receptors that activate T cells, such as Toll-like and interleukin receptors, demonstrate reduced signaling. If the receptors present on immune cells are disrupted, downstream activation and proliferation of the cell will be inhibited [47]. In space, reports have shown that spleen and thymus mass decrease post-flight [48, 49]. Furthermore, microgravity impedes proper adhesion of mononuclear cells. Suppression of surface adhesion molecules, such as CD62L and HLA-DR, prevents cell-to-cell signaling, tissue migration, and antigen presentation, dampening the overall immune response [50].

One proposed method of improving immune responses during spaceflight is the inclusion of adjuvants, such as CpG, during immunization. On Earth, CpG, an immune-stimulating molecule, acts as an agonist for Toll-like receptor 9 (TLR9), which promotes the activation of B cells, leading to a more robust immune reaction [51–53]. TLR9 stimulation can activate IRF7 signaling, which regulates T cells through IFNα release [54]. This effect appears to occur in our model following immunization with CpG alone. Alternatively, TLR9 activation can induce NF-κB which leads to inflammation [55]. The response triggered by the interaction between TLR9 and NF-κB on Earth has been reported to induce inflammation in multiple organ systems, including the lungs [56]. TLR9 is also known to be expressed by cardiomyocytes [57]. Once a cardiomyocyte responds to TLR9 signaling, pro-inflammatory cytokines are released, and a domino effect activates inflammation in neighboring cardiomyocytes via stimulation of the NF-κB pathway [58, 59]. The subsequent cytokine release activates resident macrophages [60]. The cardiomyocyte-specific inflammatory response leads to cardiac remodeling [61].

Our findings suggest that activation of pro-inflammatory pathways, in addition to those associated with TLR9, can occur when tetanus toxoid + CpG is administered in the spaceflight environment. TLR7, 8 and 13 transcripts were significantly elevated in our model. Although TLR13 has no analog in humans, TLR7 and TLR8 are present in humans and can be identified on antigen-presenting cells, such as macrophages and dendritic cells, as well as on cardiomyocytes [62–66]. TLR7 recognizes pathogens and initiates an immune response by promoting pro-inflammatory cytokine production and consequently, TLR7 antagonists inhibit cardiomyocyte inflammation [67]. TLR signaling leads to myeloid differentiation primary response 88 (MyD88) and TIR domain containing adaptor molecule 1 (TICAM-1) activation. MyD88 activation, utilized by all TLRs except TLR3, induces NF-κB, leading to the production of inflammatory cytokines [68]. In cardiomyocytes, expression of TLR8 and its interaction with MyD88 can initiate and maintain a chronic immune response in the heart [64].

Macrophages may also play a role in cardiac inflammation through cytokine release and infiltration into cardiovascular tissue. Activated resident or infiltrating cardiac macrophages can undergo cytoskeletal remodeling, as identified in flight-immunized mice, which may be attributed to tubulin re-organization [69]. Plectin (Plec) transcripts were upregulated almost 15,000-fold in flight-immunized mice. Plec proteins are involved in the organization and stabilization of the cytoskeleton and the regulation of cell adhesion, migration, and differentiation [70]. When activated, macrophages become plectin-positive [71, 72]. Similarly, allograft inflammatory factor 1 (AIF1), another marker of inflammation which is primarily expressed by macrophages, alters the cytoskeleton and was elevated in our model [73]. AIF1 interacts with TLR2 and TLR4, which were enriched in our study. These TLRs are important mediators of inflammatory pathways [74]. AIF1 also plays a role in the activation of the transcription factor the NF-κB which can induce a potent inflammatory response [75]. Prolonged activation of NF-κB can lead to tissue damage [76].

Inflammatory markers were not induced in the heart by the spaceflight environment in the absence of immunization [11], nor were they elevated in the mice which were immunized on the ground. However, inflammation associated with elevated expression of NF-κB was identified in the hearts of mice that received the TT + CpG vaccination in space. In this study, immunization can be viewed as a two-hit stressor on the heart, where one hit is represented by the vaccine itself, and the second hit by the spaceflight environment. Under these conditions, the combination of two hits in space versus one hit on Earth influences the cellular response, impacting the activation of signaling pathways and leading to inflammation. On Earth, TNF-α and IL-1β activate the canonical NF-κB-dependent inflammatory pathway [55]. In our study, these inflammatory signaling molecules were not activated in the hearts of immunized mice either on Earth or in space. In mice which were immunized in space, IL-17A and IL-22 were induced, resulting in activation of NF-κB which is capable of initiating a positive feedback loop by sustaining the release of additional pro-inflammatory cytokines [77]. This response was not evident in the mice immunized on the ground. Additionally, prolonged activation of STAT3, for example, can lead to inflammatory disease [78]. In the heart, when STAT3 is elevated in the presence of IL-6, a positive feedback loop maintains STAT3 in the phosphorylated state for extended periods [79]. This effect is regulated by suppressor of cytokine signaling 3 (SOCS3), whose activity may differ in the spaceflight environment [79]. Prolonged STAT3 activation leads to cardiac dysfunction [79, 80]. The immune response may be prolonged in flight mice due to continued activation of regulators of STAT3. The spaceflight environment impacts the immune system as a consequence of both microgravity and radiation [42, 81]. The role of NF-κB and the mechanisms by which inflammation occurs in various organs during spaceflight are currently under investigation in several laboratories [82, 83]. Interestingly, during a typical inflammatory response on Earth, NF-κB activates pro-inflammatory cytokines IL-1, IL-2, IL-6, IL-8, IL-12 and TNFα [55]. However, recent studies show that in some cases, the selective activation of cytokines, such as IFNγ, IL-6, and IL-17, during spaceflight depends on the antigen and/or adjuvant administered [84]. Furthermore, an inflammatory response can be initiated by resident and systemic cells, and the response may be tissue-specific. In the heart, inflammation can disrupt the network of microtubules and intermediate filaments in cardiomyocytes that alter the extracellular matrix and cytoskeleton [85, 86]. If inflammation becomes chronic after stress, it can direct cardiomyocytes toward ventricular remodeling [61, 87]. This would be consistent with the idea that the prolonged stress due to spaceflight, combined with the increase in inflammation after exposure to an antigen, could lead to cardiac remodeling in astronauts.

Interestingly, immunization in space induced transcripts that function to protect against oxidative stress in the presence of inflammation, including HGF, Gpx4, and TRPM2. HGF is expressed during myocardial injury and protects cardiomyocytes from oxidative stress-mediated apoptosis via inhibition of caspase activation [34, 88]. Glutathione peroxidase 4 (Gpx4), one of the most highly expressed oxidative stress-related transcripts in flight-immunized mice and a key antioxidant agent, inhibits ferroptosis through the reduction of Glutathione [89, 90]. The expression of this gene stabilizes mitochondrial membrane potential [91]. TRPM2 is an ion channel which is activated during periods of oxidative stress and helps to maintain the balance of ions in the heart [92, 93]. TRPM2 plays a vital role in protecting the heart from damage caused by reactive oxygen species [92, 93]. Cytoprotective transcripts, which are elevated in the heart in space, may contribute to maintaining homeostasis in the cardiac environment. DNA replication (Figure S2), which was elevated in our study, may reflect an increase in cardiomyocyte nuclei number in the absence of cell division which has been reported in association with improved cell responses and survival during stress exposure [94, 95]. Cardiac protection can aid in adaptation and may account for the absence of reported long-term adverse effects on the heart following space travel.

Spaceflight studies include limitations such as the number of animals which can be housed on the ISS and the number of variables that can be tested at any one time. Consequently, our study was limited to the use of same-sex, same-strain mice. Future work using the experimental design reported here should include both male and female mice to address this question further in the spaceflight environment, with and without immunization. The use of inbred mice and a defined immunization schedule over a specified amount of time should allow for reproducibility of the current findings and an expansion of the information gained here in mice of both sexes.

## Conclusion

A 30-day exposure to the spaceflight environment, combined with immunological events, such as vaccination or exposure to a pathogen, can induce inflammation and cytoskeletal remodeling in the mouse heart. Protective transcripts are induced in the heart, which reduce oxidative stress associated with inflammatory responses and prevent cardiomyocyte loss. This report lends insight into the cardiovascular response to immune challenges in space and provides a foundation for future studies designed to elucidate whether inflammation in the heart is a transient or chronic effect following vaccination in the spaceflight environment.

## Electronic supplementary material

Below is the link to the electronic supplementary material.

Supplementary Material 1

## Data Availability

The data presented in this study will be openly available and can be accessed in the NCBI Gene Expression Omnibus (GEO; http://www.ncbi.nlm.nih.gov/geo/) under accession numbers GSE223803 and GSE268421.

## Abbreviations

AIF: Allograft Inflammatory Factor
CpG: Cytidine–Phosphate–Guanosine Oligodeoxynucleotide
DNA: Deoxyribonucleic Acid
FC: Fold Change
FDR: False Discovery Rate
FPKM: Fragments per Kilobase of Transcript per Million Mapped Reads
GO: Gene Ontology
GPX: Glutathione Peroxide
GSEA: Gene Set Enrichment Analysis
HGF: Hepatocyte Growth Factor
IFNα: Interferon Alpha
IFNy: Interferon Gamma
IL: Interleukin
IP: Intraperitoneal
IPA: Ingenuity Pathway Analysis
IRF: Interferon Regulatory Factor
ISS: International Space Station
KSC: Kennedy Space Center
MyD88: Myeloid Differentiation Primary Response 88
NF-kB: Nuclear Factor Kappa B
Plec: Plectin
PTGS: Prostaglandin-Endoperoxide Synthase
RNA: Ribonucleic Acid
ROS: Reactive Oxygen Species
SIRPA: Signal-Regulator Protein Alpha
SOCS: Suppressor of Cytokine Signaling
SOD: Superoxide Dismutase
STAT: Signal Transducer and Activator of Transcription
TDAP: Tetanus, Diphtheria, and Pertussis
TFN-α: Tumor Necrosis Factor Alpha
TICAM: TIR Domain Containing Adaptor Molecule
TLR: Toll-Like Receptor
TRPM: Transient Receptor Potential Melastatin
TT: Tetanus Toxoid
TXNRD: Thioredoxin Reductase
