# Supplementary material for: Immunization induces inflammation in the mouse heart during spaceflight
Source: BMC Genomics. 2025 Mar 10;26:229. doi: 10.1186/s12864-025-11426-y (PMC11892206; doi:10.1186/s12864-025-11426-y)
Supplement: Supplementary file 1 — Supplementary Material 1 [file 12864_2025_11426_MOESM1_ESM.docx]

**a**

**b**

**c**


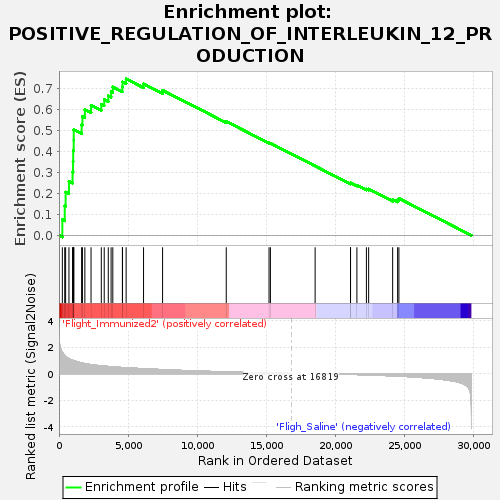


**IL-1β**

**TNF-α**

**Figure S1.** a,b) Cytokines IL-1β and TNF-α were not induced in flight mice following immunization of TT plus CpG, when compared to flight or ground controls. c) Gene Set Enrichment Analysis showed positive regulation of IL-12 production after TT+CpG vaccination in flight-immunized mice.


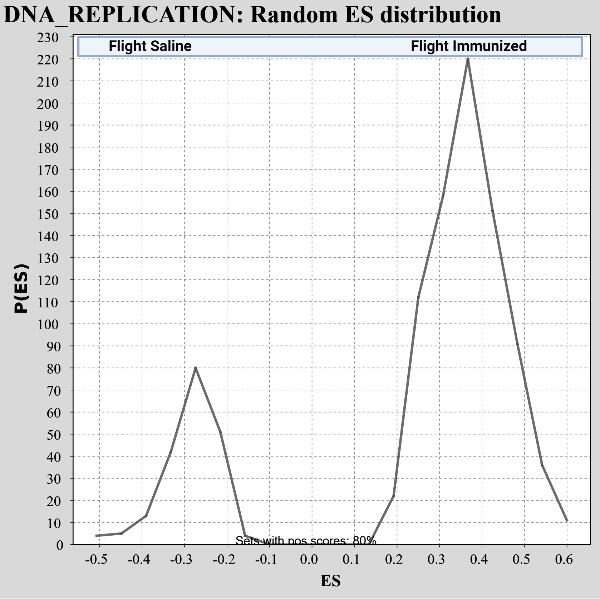


**Figure S2.** Vaccination enhanced overall DNA replication processes in flight-immunized mice compared to their in-flight saline controls.

**Table S1.** Software used for RNA Sequencing.

| Analysis item | Software | Version/date |
| --- | --- | --- |
| Quality control | FastQC | 0.10.1 |
| Adapter remove | Cutadapt | 1.10 |
| Mapping | HISAT | 2.0 |
| Transcripts assembly | StringTie | 1.3.4 |
| Differential expression analysis | DESeq2/edgeR | NA |
| GO and KEGG enrichment analysis | Perl scripts in house | NA |
| SNP/Indel analysis | samtools | 0.1.19 |
| SNP/Indel annotation | ANNOVAR | 2017.09 |
| Alternative splicing | rMATS | 4.1.1 |

**Table S2.** Weblinks used for Differential Gene Analysis following RNAseq.

| Dababase | Web links | Version/date |
| --- | --- | --- |
| Genome | <ftp://ftp.ensembl.org/pub/release-101/fasta/mus_musculus/dna/> | v101 |
| Gene Ontology (GO) | <http://geneontology.org> | 2019.05 |
| KEGG | <http://www.kegg.jp/> | 2019.05 |

**Table S3.** Primer sequences used for RT-qPCR analysis.

| Gene Name | Primer Sequence |
| --- | --- |
| NF-κB1 | Forward GCAGATGGCCCATACCTTCAA  Reverse TGGCAAATTTTGACCTGTGGGT |
| STAT3 | Forward GCAAAGAGTCACATGCCACGTTGG  Reverse AGATACCTGCTCTGCAGAAACTGC |
| IFNγ | Forward GCCACGGCACAGTCATTGA  Reverse TGCTGATGGCCTGATTGTCTT |
| IL-17A | Forward TCAGCGTGTCCAAACACTGAG  Reverse CGCCAAGGGAGTTAAAGACTT |
| IL22RA2 | Forward AAGCATTGCCTTCTAGGTCTC  Reverse TCAGAGATACACGAGCTGGTT |
| HGF | Forward CACGAACACAGCTTTTTGCC  Reverse TGATCCCAGCGCTGACAAAT |
| IL-1β | Forward GAAATGCCACCTTTTGACAGTG  Reverse TGGATGCTCTCATCAGGACAG |
| GPX3 | Forward ATCCTGCCTTCTGTCCCTGCTC  Reverse TGGTGAGGGCTCCATACTCGTA |
| SOD2 | Forward TAACGCGCAGATCATGCAGCTG  Reverse AGGCTGAAGAAGCGACCTGAGTT |
| PTGS2 | Forward GCGACATACTCAAGCAGGAGCA  Reverse AGTGGTAACCGCTCAGGTGTTG |
| TLR9 | Forward CTCCAACCGTATCCACCACC  Reverse GAGAAGTGCAGGGGGCTAAG |

**Table S4.** Ingenuity Pathway Analysis of transcripts predicting activation of NF-kB pro-inflammatory pathways. Flight TT+CpG vs. Flight Saline.

| Gene Name | Fold Change | P-Value | Regulation | Significant |
| --- | --- | --- | --- | --- |
| Il1rl2 | 6011.36 | 0.00 | up | yes |
| Per1 | 15181.82 | 0.00 | up | yes |
| Ptger1 | 1562.06 | 0.00 | up | yes |
| Per1 | 13634.71 | 0.00 | up | yes |
| Serpinb9 | 19748.95 | 0.00 | up | yes |
| Map2k3 | 15276.84 | 0.00 | up | yes |
| Rbpj | 10818.36 | 0.00 | up | yes |
| Rbpj | 9691.41 | 0.00 | up | yes |
| Sirpa | 4633.60 | 0.00 | up | yes |
| Ciita | 3137.95 | 0.00 | up | yes |
| Pik3cd | 2362.34 | 0.00 | up | yes |
| Relb | 7299.72 | 0.00 | up | yes |
| Adam8 | 2146.61 | 0.00 | up | yes |
| Afap1l2 | 3373.59 | 0.00 | up | yes |
| Cdk19 | 623.87 | 0.00 | up | yes |
| Cuedc2 | 516.46 | 0.00 | up | yes |
| Cxcl9 | 3.38 | 0.00 | up | yes |
| Il18rap | 4.94 | 0.00 | up | yes |
| Pld4 | 3.07 | 0.00 | up | yes |
| Lilrb4a | 4.88 | 0.00 | up | yes |
| Casp12 | 3.57 | 0.00 | up | yes |
| Aif1 | 3.75 | 0.00 | up | yes |
| Havcr2 | 4.22 | 0.00 | up | yes |
| Cxcr3 | 4.15 | 0.00 | up | yes |
| Gpx4 | 5706.76 | 0.00 | up | yes |
| Fpr1 | 31.51 | 0.00 | up | yes |
| Dnase1l3 | 8.95 | 0.00 | up | yes |
| C4b | 3.21 | 0.00 | up | yes |
| Adam17 | 2344.77 | 0.00 | up | yes |
| C3 | 2.08 | 0.00 | up | yes |
| Plp1 | 5.00 | 0.00 | up | yes |
| Smpdl3b | 2.47 | 0.00 | up | yes |
| Mgll | 2.19 | 0.00 | up | yes |
| Sharpin | 1508.99 | 0.00 | up | yes |
| Tlr8 | 3.42 | 0.00 | up | yes |
| Siglece | 5.15 | 0.00 | up | yes |
| Itgb2 | 2.87 | 0.00 | up | yes |
| Rps19 | 2.37 | 0.00 | up | yes |
| Tnfaip8l2 | 2.55 | 0.00 | up | yes |
| Il18 | 1364.47 | 0.00 | up | yes |
| Ctss | 3.37 | 0.00 | up | yes |
| Sbno2 | 355.26 | 0.00 | up | yes |
| Tlr12 | 6.15 | 0.00 | up | yes |
| Tlr11 | 4.45 | 0.00 | up | yes |
| Il18r1 | 6.51 | 0.00 | up | yes |
| Dab2ip | 102.91 | 0.00 | up | yes |
| H2-Eb1 | 6.72 | 0.00 | up | yes |
| H2-Eb1 | 25.87 | 0.00 | up | yes |
| H2-Aa | 6.66 | 0.00 | up | yes |
| Gnas | 13219.11 | 0.00 | up | yes |
| H2-DMb1 | 6.37 | 0.00 | up | yes |
| P2ry2 | 5623.99 | 0.00 | up | yes |
| H2-Ab1 | 7.19 | 0.00 | up | yes |
| Nfatc1 | 1353.24 | 0.00 | up | yes |
| Camk2b | 4257.93 | 0.00 | up | yes |
| Nfatc1 | 987.73 | 0.00 | up | yes |
| H2-DMa | 4.77 | 0.00 | up | yes |
| Stat1 | 4.59 | 0.00 | up | yes |
| H2-Ab1 | 9.84 | 0.00 | up | yes |
| Trpv2 | 5.98 | 0.00 | up | yes |
| H2-Aa | 17.90 | 0.00 | up | yes |
| H2-Ab1 | 4.11 | 0.00 | up | yes |
| Il2rg | 1842.99 | 0.00 | up | yes |
| Tlr3 | 0.00 | 0.00 | down | yes |
| Cyld | 0.02 | 0.00 | down | yes |
| Kars | 0.01 | 0.00 | down | yes |
| Dab2ip | 0.00 | 0.00 | down | yes |
| Adcy7 | 0.00 | 0.00 | down | yes |
| Abr | 0.00 | 0.00 | down | yes |
| Rps6ka4 | 0.00 | 0.00 | down | yes |
| Pde5a | 0.00 | 0.00 | down | yes |
| Nfkbiz | 0.00 | 0.00 | down | yes |
| Tlr3 | 0.00 | 0.00 | down | yes |
| Tlr7 | 0.00 | 0.00 | down | yes |
| Ackr2 | 0.00 | 0.00 | down | yes |
| Mecom | 0.00 | 0.00 | down | yes |
| Kng1 | 0.00 | 0.00 | down | yes |
| Relb | 0.00 | 0.00 | down | yes |
| Mapk10 | 0.00 | 0.00 | down | yes |
| Cyp2c29 | 0.00 | 0.00 | down | yes |
| Prkacb | 0.00 | 0.00 | down | yes |
| H2-DMb2 | 0.00 | 0.00 | down | yes |
| Camk2b | 0.00 | 0.00 | down | yes |

**Table S5.** Upregulated transcripts involved in the NF-kB pro-inflammatory pathway, as identified by Ingenuity Pathway Analysis. Fold Changes reflect gene expression in Flight TT+CpG immunized groups vs. Flight Saline controls.

| NFKB Network | | |
| --- | --- | --- |
| Symbol | **Expr Fold Change** | **Expr p-value** |
| ADAM8 | 2146.613 | 4.75E-08 |
| C3 | 2.078755 | 6.11E-05 |
| ITGB2 | 2.868409 | 0.000197 |
| P2RY2 | 5623.987 | 1.41E-08 |
| PLD4 | 3.070152 | 4.67E-06 |
| SIRPA | 4633.603 | 6.79E-09 |
| ADAM17 | 2344.767 | 4.25E-05 |
| AFAP1L2 | 3373.587 | 6.1E-08 |
| CAMK2B | 4257.933 | 7.27E-08 |
| NFATC1 | 1353.243 | 5.43E-08 |
| PIK3CD | 2362.34 | 1.61E-08 |
| RBPJ | 10818.36 | 1.97E-09 |
| RELB | 7299.72 | 1.65E-08 |

**Table S6.** Gene Set Enrichment Analysis identified transcripts that upregulate NF-kB signaling.

| **SYMBOL** | **RANK IN GENE LIST** | **RANK METRIC SCORE** | **RUNNING ES** | **CORE ENRICHMENT** |
| --- | --- | --- | --- | --- |
| [Bcl2a1b](https://ensembl.org/Search/Results?q=Bcl2a1b) | 71 | 2.077 | 0.0420 | Yes |
| [Prkcb](https://ensembl.org/Search/Results?q=Prkcb) | 88 | 1.991 | 0.0841 | Yes |
| [Ccl4](https://ensembl.org/Search/Results?q=Ccl4) | 215 | 1.638 | 0.1149 | Yes |
| [Ltb](https://ensembl.org/Search/Results?q=Ltb) | 242 | 1.583 | 0.1478 | Yes |
| [Zap70](https://ensembl.org/Search/Results?q=Zap70) | 347 | 1.440 | 0.1752 | Yes |
| [Btk](https://ensembl.org/Search/Results?q=Btk) | 361 | 1.423 | 0.2051 | Yes |
| [Tnfsf14](https://ensembl.org/Search/Results?q=Tnfsf14) | 732 | 1.118 | 0.2166 | Yes |
| [Traf1](https://ensembl.org/Search/Results?q=Traf1) | 745 | 1.112 | 0.2400 | Yes |
| [Vcam1](https://ensembl.org/Search/Results?q=Vcam1) | 762 | 1.103 | 0.2631 | Yes |
| [Gadd45b](https://ensembl.org/Search/Results?q=Gadd45b) | 766 | 1.101 | 0.2865 | Yes |
| [Bcl2](https://ensembl.org/Search/Results?q=Bcl2) | 891 | 1.035 | 0.3045 | Yes |
| [Ticam1](https://ensembl.org/Search/Results?q=Ticam1) | 906 | 1.031 | 0.3260 | Yes |
| [Icam1](https://ensembl.org/Search/Results?q=Icam1) | 930 | 1.018 | 0.3470 | Yes |
| [Plcg2](https://ensembl.org/Search/Results?q=Plcg2) | 942 | 1.014 | 0.3683 | Yes |
| [Blnk](https://ensembl.org/Search/Results?q=Blnk) | 999 | 0.996 | 0.3878 | Yes |
| [Tnfsf13b](https://ensembl.org/Search/Results?q=Tnfsf13b) | 1053 | 0.973 | 0.4068 | Yes |
| [Prkcq](https://ensembl.org/Search/Results?q=Prkcq) | 1078 | 0.966 | 0.4266 | Yes |
| [Card11](https://ensembl.org/Search/Results?q=Card11) | 1159 | 0.940 | 0.4441 | Yes |
| [Lck](https://ensembl.org/Search/Results?q=Lck) | 1272 | 0.902 | 0.4596 | Yes |
| [Nfkbia](https://ensembl.org/Search/Results?q=Nfkbia) | 1686 | 0.786 | 0.4625 | Yes |
| [Tlr4](https://ensembl.org/Search/Results?q=Tlr4) | 1696 | 0.784 | 0.4790 | Yes |
| [Tradd](https://ensembl.org/Search/Results?q=Tradd) | 1764 | 0.769 | 0.4932 | Yes |
| [Ddx58](https://ensembl.org/Search/Results?q=Ddx58) | 1801 | 0.762 | 0.5083 | Yes |
| [Cd14](https://ensembl.org/Search/Results?q=Cd14) | 1939 | 0.734 | 0.5194 | Yes |
| [Ticam2](https://ensembl.org/Search/Results?q=Ticam2) | 1976 | 0.727 | 0.5337 | Yes |
| [Birc3](https://ensembl.org/Search/Results?q=Birc3) | 2076 | 0.706 | 0.5455 | Yes |
| [Bcl2a1d](https://ensembl.org/Search/Results?q=Bcl2a1d) | 2128 | 0.697 | 0.5587 | Yes |
| [Ccl19](https://ensembl.org/Search/Results?q=Ccl19) | 2320 | 0.667 | 0.5665 | Yes |
| [Gm13304](https://ensembl.org/Search/Results?q=Gm13304) | 2378 | 0.660 | 0.5787 | Yes |
| [Ripk1](https://ensembl.org/Search/Results?q=Ripk1) | 2408 | 0.654 | 0.5917 | Yes |
| [Bcl2a1a](https://ensembl.org/Search/Results?q=Bcl2a1a) | 2868 | 0.599 | 0.5891 | Yes |
| [Bcl2l1](https://ensembl.org/Search/Results?q=Bcl2l1) | 3313 | 0.552 | 0.5860 | Yes |
| [Lyn](https://ensembl.org/Search/Results?q=Lyn) | 3356 | 0.548 | 0.5963 | Yes |
| [Ccl21b](https://ensembl.org/Search/Results?q=Ccl21b) | 3614 | 0.525 | 0.5989 | Yes |
| [Relb](https://ensembl.org/Search/Results?q=Relb) | 3939 | 0.499 | 0.5987 | Yes |
| [Map3k14](https://ensembl.org/Search/Results?q=Map3k14) | 3981 | 0.497 | 0.6080 | Yes |
| [Traf2](https://ensembl.org/Search/Results?q=Traf2) | 4406 | 0.465 | 0.6036 | Yes |
| [Cd40](https://ensembl.org/Search/Results?q=Cd40) | 4601 | 0.452 | 0.6068 | Yes |
| [Cflar](https://ensembl.org/Search/Results?q=Cflar) | 4691 | 0.446 | 0.6134 | Yes |
| [Syk](https://ensembl.org/Search/Results?q=Syk) | 4861 | 0.434 | 0.6170 | Yes |

**Table S7.** Upregulated transcripts involved in the IFNy pro-inflammatory pathway, identified by Ingenuity Pathway Analysis. Fold Changes reflect gene expression in Flight TT+CpG immunized groups vs. Flight Saline controls.

| IFNy Network | | |  |
| --- | --- | --- | --- |
| Symbol | **Expr Fold Change** | **Expr p-value** | |
| C4A/C4B | 3.206542607 | 4.11519E-05 | |
| CIITA | 3137.953333 | 8.72221E-09 | |
| CTSS | 3.369871405 | 0.000380747 | |
| Cxcl9 | 3.382658251 | 6.74257E-07 | |
| CXCR3 | 4.149268993 | 1.77142E-05 | |
| HAVCR2 | 4.216796725 | 1.1316E-05 | |
| HLA-DQA1 | 17.90108678 | 0.00032122 | |
| HLA-DQB1 | 9.842891018 | 0.000145668 | |
| IL18R1 | 6.514045867 | 0.000676318 | |
| IL2RG | 1842.986667 | 0.000598997 | |
| LILRB4 | 4.883295695 | 6.26441E-06 | |
| PLP1 | 5.002745213 | 8.10534E-05 | |
| SMPDL3B | 2.468937389 | 0.000115435 | |
| STAT1 | 4.59174743 | 1.27914E-05 | |
| TRPV2 | 5.982629634 | 0.000276269 | |
| Casp12 | 3.573261058 | 6.38538E-06 | |
| GPX4 | 5706.76 | 2.3862E-05 | |
| HLA-DMA | 4.77443783 | 1.89474E-06 | |
| HLA-DMB | 6.369950419 | 1.15299E-08 | |
| HLA-DRB5 | 25.87200296 | 1.74663E-09 | |
| IL18 | 1364.473333 | 0.000352468 | |
| SBNO2 | 355.2633333 | 0.000407708 | |
| SERPINB9 | 19748.95333 | 1.12472E-09 | |
| SHARPIN | 1508.99 | 0.000153306 | |
| TLR8 | 3.419024279 | 0.000186238 | |

**Table S8.** Gene Set Enrichment Analysis identified transcripts that promote IFNy activation.

| **SYMBOL** | | **RANK IN GENE LIST** | **RANK METRIC SCORE** | | **RUNNING ES** | **CORE ENRICHMENT** |
| --- | --- | --- | --- | --- | --- | --- |
| [Klrk1](https://ensembl.org/Search/Results?q=Klrk1) | 49 | | | 2.177 | 0.0486 | Yes |
| [Havcr2](https://ensembl.org/Search/Results?q=Havcr2) | 65 | | | 2.097 | 0.0964 | Yes |
| [Tlr7](https://ensembl.org/Search/Results?q=Tlr7) | 133 | | | 1.826 | 0.1363 | Yes |
| [Slc11a1](https://ensembl.org/Search/Results?q=Slc11a1) | 157 | | | 1.771 | 0.1763 | Yes |
| [Ptpn22](https://ensembl.org/Search/Results?q=Ptpn22) | 207 | | | 1.652 | 0.2128 | Yes |
| [Klre1](https://ensembl.org/Search/Results?q=Klre1) | 343 | | | 1.448 | 0.2417 | Yes |
| [Sash3](https://ensembl.org/Search/Results?q=Sash3) | 348 | | | 1.440 | 0.2747 | Yes |
| [Il18r1](https://ensembl.org/Search/Results?q=Il18r1) | 393 | | | 1.394 | 0.3054 | Yes |
| [Tlr8](https://ensembl.org/Search/Results?q=Tlr8) | 418 | | | 1.360 | 0.3360 | Yes |
| [H2-M3](https://ensembl.org/Search/Results?q=H2-M3) | 479 | | | 1.289 | 0.3637 | Yes |
| [Cd226](https://ensembl.org/Search/Results?q=Cd226) | 550 | | | 1.226 | 0.3896 | Yes |
| [Cd27](https://ensembl.org/Search/Results?q=Cd27) | 602 | | | 1.192 | 0.4154 | Yes |
| [Il12rb1](https://ensembl.org/Search/Results?q=Il12rb1) | 679 | | | 1.145 | 0.4392 | Yes |
| [Ulbp1](https://ensembl.org/Search/Results?q=Ulbp1) | 755 | | | 1.107 | 0.4622 | Yes |
| [Cd160](https://ensembl.org/Search/Results?q=Cd160) | 849 | | | 1.058 | 0.4835 | Yes |
| [Il18](https://ensembl.org/Search/Results?q=Il18) | 914 | | | 1.028 | 0.5051 | Yes |
| [Il23a](https://ensembl.org/Search/Results?q=Il23a) | 982 | | | 1.003 | 0.5259 | Yes |
| [Irf8](https://ensembl.org/Search/Results?q=Irf8) | 1027 | | | 0.985 | 0.5472 | Yes |
| [Tlr9](https://ensembl.org/Search/Results?q=Tlr9) | 1065 | | | 0.972 | 0.5683 | Yes |
| [Cd3e](https://ensembl.org/Search/Results?q=Cd3e) | 1072 | | | 0.969 | 0.5905 | Yes |
| [Arid5a](https://ensembl.org/Search/Results?q=Arid5a) | 1136 | | | 0.948 | 0.6102 | Yes |
| [Cd2](https://ensembl.org/Search/Results?q=Cd2) | 1349 | | | 0.873 | 0.6232 | Yes |
| [Il27ra](https://ensembl.org/Search/Results?q=Il27ra) | 1556 | | | 0.814 | 0.6351 | Yes |
| [Tnfsf9](https://ensembl.org/Search/Results?q=Tnfsf9) | 1638 | | | 0.797 | 0.6508 | Yes |
| [Tlr4](https://ensembl.org/Search/Results?q=Tlr4) | 1696 | | | 0.784 | 0.6670 | Yes |
| [Cd14](https://ensembl.org/Search/Results?q=Cd14) | 1939 | | | 0.734 | 0.6758 | Yes |
| [Ticam2](https://ensembl.org/Search/Results?q=Ticam2) | 1976 | | | 0.727 | 0.6913 | Yes |
| [Rasgrp1](https://ensembl.org/Search/Results?q=Rasgrp1) | 2429 | | | 0.652 | 0.6912 | Yes |
| [Ccr2](https://ensembl.org/Search/Results?q=Ccr2) | 2593 | | | 0.631 | 0.7003 | Yes |
| [Pycard](https://ensembl.org/Search/Results?q=Pycard) | 2800 | | | 0.608 | 0.7074 | Yes |
| [Cd244a](https://ensembl.org/Search/Results?q=Cd244a) | 2818 | | | 0.606 | 0.7208 | Yes |
| [Cebpg](https://ensembl.org/Search/Results?q=Cebpg) | 2956 | | | 0.588 | 0.7297 | Yes |

**Table S9.** Ingenuity Pathway Analysis of cytoskeletal markers: Flight TT+CpG vs. Flight Saline.

| Gene Name | Fold Change | P-Value | Regulation | Significant |
| --- | --- | --- | --- | --- |
| Septin7 | 208962.37 | 0.00 | up | yes |
| Rhobtb1 | 64660.70 | 0.00 | up | yes |
| Tuba4a | 51605.72 | 0.00 | up | yes |
| Tacc2 | 23701.50 | 0.00 | up | yes |
| Ptpn21 | 17032.83 | 0.00 | up | yes |
| Plec | 14973.05 | 0.00 | up | yes |
| Frmd5 | 12906.65 | 0.00 | up | yes |
| Rbm39 | 10592.43 | 0.00 | up | yes |
| Epb41 | 9949.66 | 0.00 | up | yes |
| Dst | 9221.26 | 0.00 | up | yes |
| Kif1b | 8502.25 | 0.00 | up | yes |
| Relb | 7299.72 | 0.00 | up | yes |
| Abi1 | 6416.73 | 0.00 | up | yes |
| Ss18 | 5784.36 | 0.00 | up | yes |
| Sdccag8 | 5568.34 | 0.00 | up | yes |
| Inppl1 | 4967.63 | 0.00 | up | yes |
| Ttbk2 | 4821.85 | 0.00 | up | yes |
| Npm1 | 4821.70 | 0.00 | up | yes |
| Sirpa | 4633.60 | 0.00 | up | yes |
| Arhgef10l | 4416.19 | 0.00 | up | yes |
| Pkp4 | 4386.32 | 0.00 | up | yes |
| Trmt10a | 4367.62 | 0.00 | up | yes |
| Camk2b | 4257.93 | 0.00 | up | yes |
| Mad1l1 | 4140.09 | 0.00 | up | yes |
| Map2 | 3965.95 | 0.00 | up | yes |
| Dvl2 | 3558.26 | 0.00 | up | yes |
| Nsmf | 3317.67 | 0.00 | up | yes |
| Jade1 | 2994.82 | 0.00 | up | yes |
| Cep112 | 2772.76 | 0.00 | up | yes |
| Csrp1 | 2622.66 | 0.00 | up | yes |
| Zmym6 | 2594.28 | 0.00 | up | yes |
| Dnm1 | 2593.32 | 0.00 | up | yes |
| Cep295 | 2459.66 | 0.00 | up | yes |
| Pik3cd | 2362.34 | 0.00 | up | yes |
| Adam17 | 2344.77 | 0.00 | up | yes |
| Lrrc45 | 2051.22 | 0.00 | up | yes |
| Fhod3 | 1488.22 | 0.00 | up | yes |
| Tapt1 | 1473.40 | 0.00 | up | yes |
| Gpm6b | 1412.19 | 0.00 | up | yes |
| Fam110b | 1409.62 | 0.00 | up | yes |
| Mical3 | 1314.28 | 0.00 | up | yes |
| Pak1 | 1308.23 | 0.00 | up | yes |
| Inpp5d | 1019.05 | 0.00 | up | yes |
| Amph | 978.32 | 0.00 | up | yes |
| Wdr90 | 944.57 | 0.00 | up | yes |
| Trpm2 | 838.01 | 0.00 | up | yes |
| Ttn | 835.15 | 0.00 | up | yes |
| Ank3 | 798.04 | 0.00 | up | yes |
| Armc9 | 665.88 | 0.00 | up | yes |
| Spag4 | 562.82 | 0.00 | up | yes |
| Arhgef6 | 514.26 | 0.00 | up | yes |
| Frmpd3 | 444.93 | 0.00 | up | yes |
| Togaram1 | 70.46 | 0.00 | up | yes |
| Ttll5 | 39.93 | 0.00 | up | yes |
| Tnnt2 | 39.03 | 0.00 | up | yes |
| Rapsn | 31.93 | 0.00 | up | yes |
| Cryab | 17.25 | 0.00 | up | yes |
| Nubp2 | 14.18 | 0.00 | up | yes |
| Insrr | 11.61 | 0.00 | up | yes |
| Ablim1 | 9.04 | 0.00 | up | yes |
| Limch1 | 6.60 | 0.00 | up | yes |
| Septin3 | 5.85 | 0.00 | up | yes |
| Misp | 4.98 | 0.00 | up | yes |
| Smtnl2 | 4.80 | 0.00 | up | yes |
| Stoml2 | 4.47 | 0.00 | up | yes |
| Fry | 4.15 | 0.00 | up | yes |
| Coro1a | 3.82 | 0.00 | up | yes |
| Aif1 | 3.75 | 0.00 | up | yes |
| Clip1 | 3.53 | 0.00 | up | yes |
| Itgb7 | 3.25 | 0.00 | up | yes |
| Gbp2 | 3.18 | 0.00 | up | yes |
| Ccdc88b | 3.04 | 0.00 | up | yes |
| Myo1g | 2.93 | 0.00 | up | yes |
| Itgb2 | 2.87 | 0.00 | up | yes |
| Vav1 | 2.80 | 0.00 | up | yes |
| Coro1a | 2.62 | 0.00 | up | yes |
| Fmnl1 | 2.41 | 0.00 | up | yes |
| Mid1ip1 | 2.29 | 0.00 | up | yes |
| Was | 2.15 | 0.00 | up | yes |
| Rabgap1 | 0.49 | 0.00 | down | yes |
| Son | 0.45 | 0.00 | down | yes |
| Ptp4a1 | 0.44 | 0.00 | down | yes |
| Ttc17 | 0.24 | 0.00 | down | yes |
| Bbs9 | 0.21 | 0.00 | down | yes |
| Cdc14b | 0.09 | 0.00 | down | yes |
| Nav1 | 0.04 | 0.00 | down | yes |
| Ppp4r3b | 0.04 | 0.00 | down | yes |
| Pip5k1a | 0.02 | 0.00 | down | yes |
| Fn1 | 0.02 | 0.00 | down | yes |
| Cyld | 0.02 | 0.00 | down | yes |
| Lima1 | 0.01 | 0.00 | down | yes |
| Frmpd1 | 0.00 | 0.00 | down | yes |
| Phip | 0.00 | 0.00 | down | yes |
| Clasp1 | 0.00 | 0.00 | down | yes |
| Klc2 | 0.00 | 0.00 | down | yes |
| Xirp2 | 0.00 | 0.00 | down | yes |
| Brwd1 | 0.00 | 0.00 | down | yes |
| Akap5 | 0.00 | 0.00 | down | yes |
| Nin | 0.00 | 0.00 | down | yes |
| Dmd | 0.00 | 0.00 | down | yes |
| Cetn4 | 0.00 | 0.00 | down | yes |
| Rara | 0.00 | 0.00 | down | yes |
| Cetn4 | 0.00 | 0.00 | down | yes |
| Ehbp1 | 0.00 | 0.00 | down | yes |
| Pard3 | 0.00 | 0.00 | down | yes |
| Ablim1 | 0.00 | 0.00 | down | yes |
| Sorbs2 | 0.00 | 0.00 | down | yes |
| Sptbn1 | 0.00 | 0.00 | down | yes |
| Relb | 0.00 | 0.00 | down | yes |
| Hrg | 0.00 | 0.00 | down | yes |
| Arhgap39 | 0.00 | 0.00 | down | yes |
| Auts2 | 0.00 | 0.00 | down | yes |
| Itgb1 | 0.00 | 0.00 | down | yes |
| Epb41l3 | 0.00 | 0.00 | down | yes |
| Amot | 0.00 | 0.00 | down | yes |
| Synpo | 0.00 | 0.00 | down | yes |
| Efr3b | 0.00 | 0.00 | down | yes |
| Kif1b | 0.00 | 0.00 | down | yes |
| Ift122 | 0.00 | 0.00 | down | yes |
| Camk2b | 0.00 | 0.00 | down | yes |
| Dlg5 | 0.00 | 0.00 | down | yes |
| D430042O09Rik | 0.00 | 0.00 | down | yes |
| Obscn | 0.00 | 0.00 | down | yes |
| Usp33 | 0.00 | 0.00 | down | yes |
| Kng1 | 0.00 | 0.00 | down | yes |
| Nin | 0.00 | 0.00 | down | yes |
| Utrn | 0.00 | 0.00 | down | yes |
| Actr3 | 0.00 | 0.00 | down | yes |
| Rcc2 | 0.00 | 0.00 | down | yes |
| Sptan1 | 0.00 | 0.00 | down | yes |
| Trmt10a | 0.00 | 0.00 | down | yes |
| Tubd1 | 0.00 | 0.00 | down | yes |
| Myadm | 0.00 | 0.00 | down | yes |
| Phldb2 | 0.00 | 0.00 | down | yes |
| Abr | 0.00 | 0.00 | down | yes |
| Sptan1 | 0.00 | 0.00 | down | yes |
| Kif21a | 0.00 | 0.00 | down | yes |
| Sptan1 | 0.00 | 0.00 | down | yes |
| Jade1 | 0.00 | 0.00 | down | yes |
| Ivns1abp | 0.00 | 0.00 | down | yes |
| Fry | 0.00 | 0.00 | down | yes |
| Synpo2 | 0.00 | 0.00 | down | yes |
